# The Possible Cellular Mechanism for Extending Lifespan of Mice with Rapamycin

**DOI:** 10.1007/s12575-009-9015-y

**Published:** 2009-09-01

**Authors:** Shulin Li

**Affiliations:** 1 Department of Comparative Biomedical Sciences, School of Veterinary Medicine, Louisiana State University, Baton Rouge, 70803, LA, USA

## 

Cell death and survival are based on the net result of hundreds of signals and their cognate pathways. Animal survival is based on the net survival and health of cells in each tissue and organ. Although in certain circumstances some cells are programmed to die during the lifetime to maintain a healthy body, these two units of life are normally greatly synchronized, or, at the least, survival signals in both units are normally in agreement with each other. The mechanism that connects these two life units has never been explored; however, three articles recently published in Nature may establish such a connection. A group led by Richard A Miller discovered that rapamycin, a specific inhibitor of the mTOR signaling pathway, extends the lifespan in genetically heterogeneous mice in three different experimental locations [1]. Amazingly, the treatment was not initiated at an early age but in the late phase when mice were 600 days old, which relates to approximately 50 years old in humans. This phenomenal observation is not associated with a clear mechanistic revelation.

In the same volume, a group led by Rafi Ahmed discovered that inhibition of mTOR by rapamycin independently boosted the quantity and quality of memory CD8 T cells [2]. This discovery was confirmed by an RNA interference technique which knocked down the mTOR pathway. Another group led by Young Choi found that rapamycin extends memory T cell generation and survival by regulating mitochondrial fatty acid oxidation and induces AMP-activated kinase activity [3]. These increased memory T cell responses may account for the increased lifespan of the mice receiving rapamycin. This hypothesis needs to be tested.

## References

[B1] Harrison DE Strong R Sharp ZD Nelson JF Astle CM Flurkey K Nadon NL Wikinson JE Frenkel K Carter CS Pahor M Javors MA Fernandez E Miller RA Rapamycin fed late in life extends lifespan in genetically heterogenous mice Nature 2009 10.1038/nature08221 19587680 PMC2786175

[B2] Araki K Turner AP Shaffer VO Gangappa S Keller SA Bachmann MF Larsen CP Ahmed R mTOR regulates memory CD8 T-cell differentiation Nature 2009 10.1038/nature08155 19543266 PMC2710807

[B3] Pearce EL Walsh MC Cejas PF Harms GM Shen H Wang LS Jones RG Choi Y Enhancing CD8 T-cell memory by modulating fatty acid metabolism Nature 2009 10.1038/nature08097 19494812 PMC2803086

